# Poor Bone Health Associated with Reduced Cerebral Perfusion and Brain Volume in Older Adults

**DOI:** 10.3390/diagnostics16040529

**Published:** 2026-02-10

**Authors:** Tiffany Y. So, James F. Griffith, Jill Abrigo, Lin Shi, David K. W. Yeung, Jason Leung, Timothy Kwok, Vincent C. T. Mok

**Affiliations:** 1 Department of Imaging and Interventional Radiology, Prince of Wales Hospital, The Chinese University of Hong Kong, Hong Kong, China; 2 Jockey Club Centre for Osteoporosis Care and Control, The Chinese University of Hong Kong, Hong Kong, China; jason-leung@cuhk.edu.hk; 3 Department of Medicine & Therapeutics, The Chinese University of Hong Kong, Hong Kong, China; tkwok@cuhk.edu.hk (T.K.);

**Keywords:** osteoporosis, bone mineral density, vertebral perfusion, marrow fat, cognitive impairment, MRI

## Abstract

**Background:** Bone health and brain function may be closely interconnected through a complex bone–brain axis. The relationship between bone mineral density (BMD), vertebral perfusion, marrow composition, cerebral perfusion, brain volume, and cognitive decline, however, remain incompletely understood. **Methods:** Ninety-nine female subjects (mean age 65.00 ± 5.00 years) with clinically suspected mild cognitive impairment underwent dual-energy X-ray absorptiometry, carotid ultrasound, and multimodal magnetic resonance imaging (MRI) of the brain and lumbar spine to measure BMD, bone perfusion, marrow fat content as well as cerebral perfusion, cerebral volume, cerebral white matter burden and large vessel atherosclerosis. Cognitive function was assessed using the Hong Kong Montreal Cognitive Assessment (HK-MoCA). Bone, cerebral, vascular, and cognitive measures were correlated using Spearman correlation coefficients and compared in group comparisons. **Results:** Lower BMD was correlated with reduced subcortical cerebral blood flow (CBF) (r = 0.27, *p* = 0.031) and lower total brain parenchymal volume (r = 0.25, *p* = 0.021). Reduced bone marrow perfusion and increased marrow fat content were also associated with lower total brain parenchymal volume (r = 0.24, *p* = 0.023 and r = −0.26, *p* = 0.025). Subjects with the lowest L3 vertebral body perfusion or highest marrow fat content had significantly reduced total brain and hippocampal volumes (*p* = 0.029–0.049) compared with those with the highest perfusion or lowest marrow fat content. **Conclusions:** This study shows an association between lower BMD, reduced vertebral perfusion, and increased marrow fat with reduced brain parenchymal volumes and reduced brain perfusion. Further studies are warranted to clarify these relationships and explore the underlying shared mechanisms affecting bone health and cerebral microvascular and structural brain changes.

## 1. Introduction

Cognitive impairment and osteoporosis are two common, debilitating conditions in older adults, often co-occurring, and substantially impacting quality of life and independence. While traditionally considered separate entities, recent evidence suggests that bone health and brain function may be closely interconnected through a complex bone–brain axis [1,2,3].

Low bone mineral density (BMD) is associated with an increased risk of both cognitive decline and cerebrovascular disease. In a large longitudinal cohort, BMD, particularly at the cortical level, was independently and inversely associated with cognitive decline in community-dwelling older women [4]. In other studies, lower BMD correlated with cognitive impairment and greater risk of cognitive deterioration over time [5,6]. Patients with osteoporosis have an increased risk to develop a stroke compared to patients with normal BMD, independent of other risk factors [7]. Small vessel disease, including white matter changes and lacunar infarcts, are more commonly observed in subjects with osteoporosis (odds ratio 1.5–2.6) compared to normal BMD subjects [8]. Structural brain changes, including regional reductions in cerebral volume, have also been found in patients with osteoporosis [9,10]. 

Mechanistically, the bone–brain axis may be mediated by a multitude of bidirectional vascular, inflammatory, molecular and neuroendocrine pathways. Chronic inflammation and endothelial dysfunction may contribute to both impaired bone metabolism and increased cerebrovascular vulnerability [11,12,13]. Sympathetic outflow can influence osteoblast and osteoclast activity through catecholaminergic signalling, while hypothalamic–pituitary–adrenal axis activation further alters bone turnover and cerebrovascular tone [14,15,16]. Experimental work suggests that disturbances in these regulatory circuits may promote bone loss and microvascular injury, offering potential explanations for the co-occurrence of osteoporosis, cognitive impairment and small vessel disease in older adults. However, despite the expanding experimental and epidemiological literature on the bone–brain axis, comprehensive imaging studies evaluating direct markers of skeletal and cerebral status remain limited. Reduced bone perfusion and increased marrow fat content are well recognised features of reduced BMD. However, it is not known whether bone perfusion and marrow fat content are directly associated with regional cerebral perfusion or brain atrophy, or how these parameters relate to cognitive decline.

Advanced magnetic resonance imaging (MRI) techniques allow the assessment of bone perfusion and marrow fat content as well as brain perfusion and volume. Dynamic contrast-enhanced (DCE) MRI is the most widely used technique for quantifying bone marrow perfusion, while MR spectroscopy (MRS) and chemical shift encoding MRI allow the quantitative assessment of fatty marrow content. Cerebral perfusion can be measured using dynamic susceptibility contrast (DSC) MRI, a first-pass technique that allows robust quantification of perfusion following a bolus injection of a gadolinium-based contrast agent. Alternatively, arterial spin labelling (ASL), a non-contrast technique, is increasingly used to quantify whole brain and regional cerebral blood flow using magnetically labelled arterial blood as an endogenous tracer, correlating well with DSC [17]. Together with high-resolution structural MRI for volumetric brain analysis, applying such a multimodal MRI framework provides an opportunity to directly test whether bone perfusion and marrow composition in osteoporosis are linked to cerebral perfusion, brain structure and cognition, and to delineate shared microvascular and metabolic mechanisms along the brain–bone axis.

This study was therefore designed to:

• Examine the associations between BMD and cerebral perfusion, brain volume, and cognitive function in older female patients;
• Investigate whether vertebral bone perfusion and marrow fat content are associated with cerebral perfusion, brain volume, and cognitive function; and
• Assess the relationship of these parameters with markers of cerebral small and large vessel disease, given the potential involvement of both microvascular and macrovascular disease in osteoporosis and cognitive decline.

## 2. Methods

### 2.1. Subjects

Consecutive female subjects with clinically suspected mild cognitive impairment (MCI) were prospectively recruited for this study. Subjects were first identified when they presented for preliminary evaluation at the Jockey Club Centre for Disease and Control. Potential participants were then referred to the Neurology MCI clinic at our institution for comprehensive evaluation, including cognitive testing and review of medical history. To be eligible for inclusion, patients had to have no history or clinical evidence of metabolic bone disease or osseous metastases, no prior spinal or intracranial surgical intervention or drug treatment, and no known neurodegenerative or other neurological disorders that could confound assessment. Subjects were excluded if they declined intravenous contrast administration (*n* = 2) or if image quality was non-diagnostic due to excessive motion artefacts (*n* = 4). A total of 99 female subjects were included in the final analysis. The study protocol was approved by the institutional ethics review board and conducted in accordance with the principles of the Declaration of Helsinki. All subjects provided informed signed consent to participate in the study. Each subject underwent a standardised set of assessments, described in the following subsections.

### 2.2. Cognitive Function Assessment

Cognitive status was assessed using the Hong Kong version of Montreal Cognitive Assessment (HK-MoCA), a validated tool for identification of mild cognitive impairment and dementia in older Chinese adults. This test, which can be completed in 15 min, has high sensitivity and specificity for differentiating between normal, mild cognitive impairment, and dementia [18]. The assessment was undertaken by neurology clinicians who routinely perform such evaluations in clinical settings dedicated to memory and cognitive disorders. The HK-MoCA score ranges from 0 to 30, with a score of 26 or above considered indicative of normal cognitive function.

### 2.3. Dual-Energy X-Ray Absorptiometry (DEXA)

Areal bone mineral density (BMDa) was measured at the lumbar spine, right femoral neck and proximal femur (total hip) using a clinical dual-energy X-ray absorptiometry unit (Hologic QDR-4500W, Hologic Inc., Bedford, MA, USA). The lowest T-score among the measured sites was used to determine the representative BMDa in g/cm^2^ and corresponding T-score, based on local reference standards [19].

### 2.4. Carotid Ultrasound Examination

**Figure 1:**
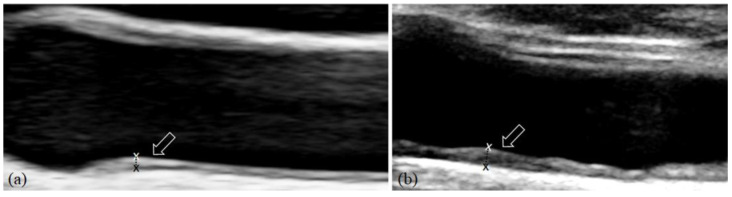
Longitudinal ultrasound of the common carotid artery. Intima-media thickness (IMT) was measured (callipers and arrowed) at the far wall of the distal portion of the common carotid arteries close to the carotid bulb. (**a**) Normal and (**b**) thickened IMT.

Carotid artery examinations were performed using a high-resolution ultrasound system (Philips Medical Systems, Bothell, WA, USA) equipped with a standard linear-array transducer. Examinations were conducted by an experienced radiologist with over 20 years of expertise in vascular ultrasound imaging. Standardised protocols were used to evaluate both common carotid arteries, with longitudinal B-mode images obtained to assess intima-media thickness (IMT) in a plaque-free segment of the distal common carotid artery. Carotid IMT was measured three times on each side at the thickest point of the far wall of the distal portion of the common carotid arteries [20] (Figure 1). For each subject, both the maximum and average IMT values of the right and left sides were recorded. The overall mean IMT was calculated by averaging the six measurements from both sides. An IMT value < 1.0 mm was considered within the normal range [20].

### 2.5. MRI Brain Image Acquisition

All brain MRI examinations were performed on a 3.0 Tesla MRI scanner (Achieva TX series, Philips Healthcare, Best, The Netherlands) using an eight-channel head-coil. The MRI protocol included the following sequences, described below and summarised in Supplementary Table S1.

#### 2.5.1. 3D T1-Weighted Imaging

Turbo field echo (TFE) sequence with repetition time (TR)/echo time (TE) = 7.4/3.4 ms, flip angle = 8°, field of view (FOV) = 250 × 250 mm^2^, 285 contiguous slices, slice thickness = 0.6 mm, and reconstruction matrix = 240 × 240.

#### 2.5.2. Axial T2-Weighted Imaging

Fat-suppressed turbo spin echo (TSE) sequence with TR/TE = 5472/80 ms, echo train length = 18, FOV = 230 × 207 mm^2^, slice thickness = 5 mm, matrix = 512 × 350, number of signal averages (NSA) = 2, and sensitivity encoding (SENSE) factor = 1.5.

#### 2.5.3. 3D Fluid-Attenuated Inversion Recovery (FLAIR)

Acquired using a 3D TSE acquisition (VISTA) in the sagittal plane with TR/TE = 8000/332 ms, inversion time = 2400 ms, field of view = 230 × 230 mm^2^, 301 contiguous slices, slice thickness = 0.55 mm, reconstruction matrix = 208 × 208, NSA = 1. Fat suppression was achieved using an inversion delay pulse of 220 ms.

#### 2.5.4. 3D Time-of-Flight MR Angiography (TOF-MRA)

**Figure 2:**
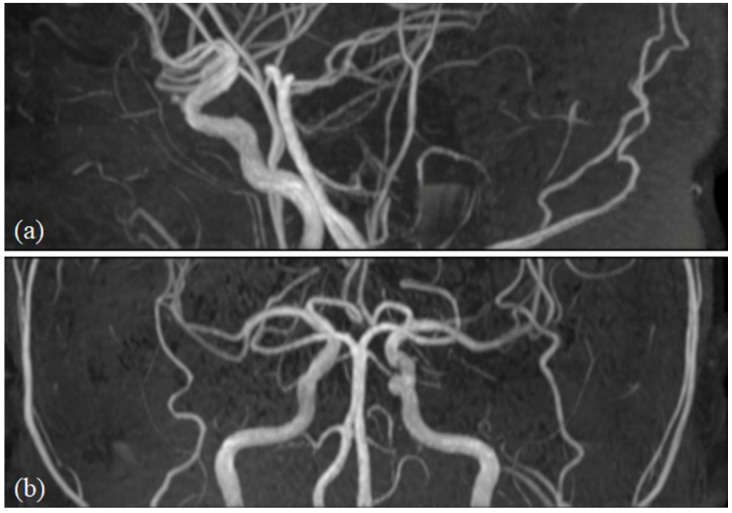
(**a**,**b**) Representative images from axial 3D time-of-flight magnetic resonance angiography (TOF-MRA) depicting the large- to medium-sized intracranial arteries.

Acquired in the axial plane with TR/TE = 25/3.5, flip angle = 20°, FOV = 180 × 180 × 84 mm^3^, voxel size, 0.3 × 0.3 × 0.7 mm^3^, to visualise the large- to medium-sized intracranial arteries (Figure 2).

#### 2.5.5. Pseudocontinous Arterial Spin Labelling (pCASL)

**Figure 3:**
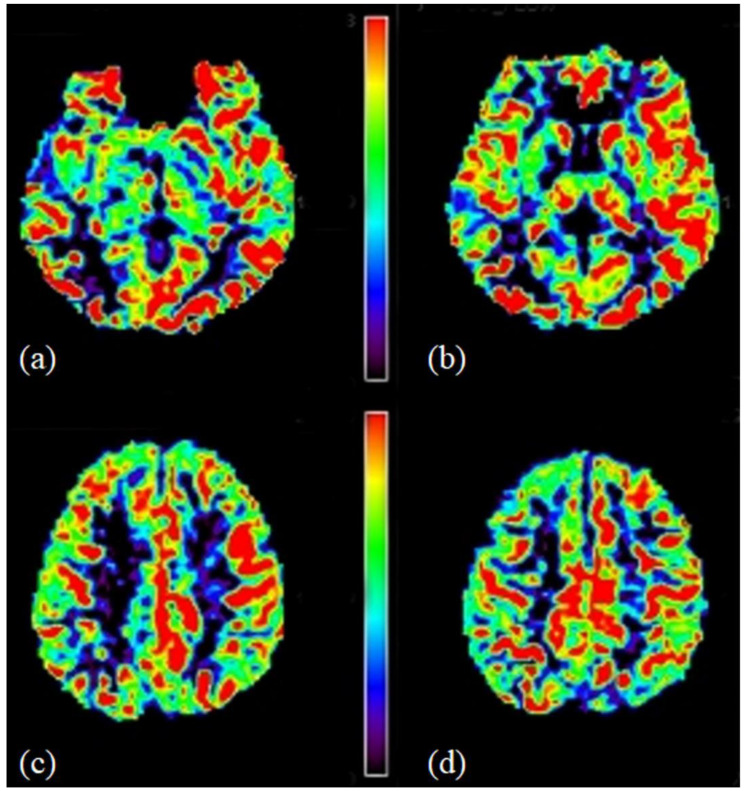
(**a**–**d**) Representative pseudocontinuous arterial spin labelling (pCASL) images at different levels showing cerebral blood flow (CBF) maps. Maps are colour-rendered against the calibration bar to reflect quantitative CBF values.

pCASL preparation consisted of labelling with a train of Hanning-shaped radiofrequency pulses (flip angle = 18°, duration = 0.5 ms, inter-pulse pause interval = 0.5 ms), applied over a total label duration of 1650 ms. Background suppression consisted of a pre-saturation pulse applied immediately before labelling and two inversion pulses at 1680 and 2830 ms. Perfusion images were acquired with a post-labelling delay (PLD) of 1525 ms using a single-shot echo-planar (EPI) technique with parallel imaging (acceleration factor = 2.5) for rapid data acquisition. Seventeen transverse sections of 7 mm thickness were acquired in ascending fashion with an in-plane resolution of 3 × 3 mm. Imaging parameters were: TR/TE = 4000/14 ms, FOV= 240 × 240 × 119 mm^3^, matrix = 80 × 79, number of label-control pairs = 38 (Figure 3).

### 2.6. MRI Brain Image Analysis

All MRI brain semiquantitative analyses were performed by a board-certified neuroradiologist with 10 years of experience in MRI brain interpretation, and all quantitative analyses were conducted by a computer scientist with over 15 years of experience of brain computational neuroimaging, both of whom were blinded to all clinical, laboratory, and cognitive data.

#### 2.6.1. White Matter Hyperintensity (WMH) Quantification

**Figure 4:**
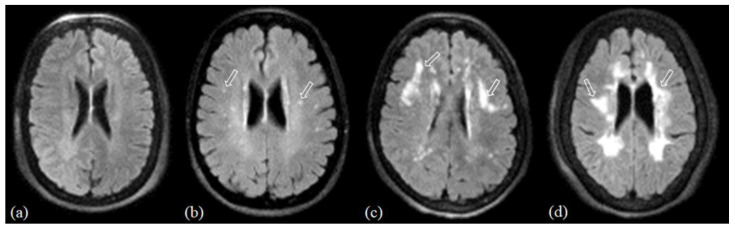
Representative axial fluid-attenuated inversion recovery (FLAIR) MR images showing increasing severity of white matter hyperintensities (WMHs; arrowed) with the corresponding assigned Fazekas grades. (**a**) No WMH, grade 0; (**b**) multiple small WMH, grade 1; (**c**) WMH with beginning confluence, grade 2; (**d**) diffuse confluent WMH, grade 3.

WMHs were evaluated on 3D FLAIR images using both semiquantitative and quantitative methods. Semiquantitative assessment of WMH burden was performed using the Fazekas scale (Figure 4): Grade 0 = none or a single punctate WMH lesion; 1 = multiple punctate lesions; 2 = beginning confluence of lesions; 3 = large confluent lesions. Quantitative WMH burden was assessed using a previously validated automated segmentation tool [21], and expressed absolute volumes.

#### 2.6.2. MRA Stenosis Scoring

Visual scoring of focal stenosis in the proximal cerebral arteries on MRA was performed using a five-point scale: 0 = no stenosis, 1 = mild (<30% stenosis), 2 = moderate (>30–60% stenosis), 3 = severe (>70% stenosis) and 4 = occluded. Carotid IMT and intracranial arterial stenosis on MRA were taken as measures of large vessel disease.

#### 2.6.3. Cerebral Perfusion

Image processing was conducted using SPM 12 (https://www.fil.ion.ucl.ac.uk/spm/) in MATLAB R2021a (MathWork Inc., Natick, MA, USA). All label and control images were first realigned to the initial control image to correct for motion. The M0 image and high-resolution T1-weighted anatomical image were then co-registered to the mean of the motion-corrected pCASL image. CBF maps were calculated using the single-compartment kinetic model recommended by the ISMRM perfusion study group [22], and spatially smoothed with an 8 mm full-width half maximum Gaussian kernel.

For regional analysis, individual T1-weighted images were segmented into tissue classes using the New Segment tool in SPM 12 (https://www.fil.ion.ucl.ac.uk/spm/), and the Automated Anatomical Labelling (AAL) atlas was inverse-warped to each subject’s native space using the fields from segmentation. The brain was parcellated into cortical lobar and deep subcortical (deep grey matter) regions, and these regions were applied to the quantified CBF maps to extract the mean CBF values.

#### 2.6.4. Brain Volume Analysis

Quantification of brain volume was performed using AccuBrain (Brainnow Medical Technology, Shenzhen, China), a fully automated segmentation tool validated for brain morphometric analysis. Volumetric 3D T1-weighted images were analysed to extract whole brain and bilateral hippocampal volumes. To account for inter-subject variability in head size, brain volumes were normalised and expressed as a fraction (%) of the total intracranial volume.

### 2.7. MRI Spine Image Acquisition

Spinal MR imaging was performed using a 3.0 Tesla whole-body MRI scanner (Achieva TX series, Philips Healthcare, Best, The Netherlands) using a phase-array spine coil. Sagittal T1-weighted (TR/TE, 450/1 ms) and T2-weighted (TR/TE, 3500/120 ms) sequences of the thoracic and lumbar spine were acquired. These images were used to exclude vertebral fractures spinal metastases, or other structural abnormalities. For quantitative assessment of bone marrow perfusion and fat content, an imaging volume of interest (VOI) was positioned at the L3 vertebral body. The L3 vertebra was selected because it provides a sufficiently large, centrally located marrow region in the mid-portion of the lumbar spine that allows reliable perfusion and single-voxel MR spectroscopy measurements without contamination from the overlying cortical bone, and is a site commonly used for such measurements [23,24]. Marrow fat content and perfusion at this level are considered representative of the entire lumbar spine and whole-body marrow composition and perfusion [25].

#### 2.7.1. DCE Perfusion Imaging

**Figure 5:**
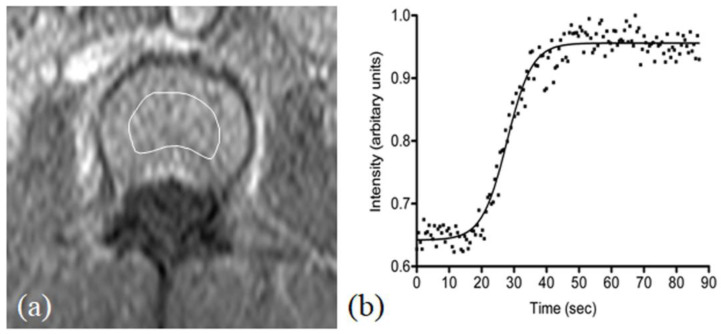
(**a**) T1-weighted axial MR image showing the region of interest (ROI) placed centrally within the L3 vertebral body for perfusion assessment. (**b**) Representative normal time–intensity perfusion curve obtained from the ROI.

Dynamic contrast-enhanced axial images were acquired through the mid-L3 vertebral body [8] (Figure 5). A total of 160 frames were acquired at a temporal resolution of 543 ms, with a total acquisition time of 1:27 min. A bolus injection of gadoteric acid (Dotarem; Guerbet, Aulnay, France) was administered at a dose of 0.15 mmol per kg body weight via an antecubital vein using a power injector (Spectris, Medrad, Inc., Indianola, PA, USA) at a rate of 2.5 mL/s, followed by a 20 mL saline flush. Dynamic scanning commenced concurrently with the contrast injection (time zero).

#### 2.7.2. Single-Voxel Proton MR Spectroscopy (1H-MRS)

**Figure 6:**
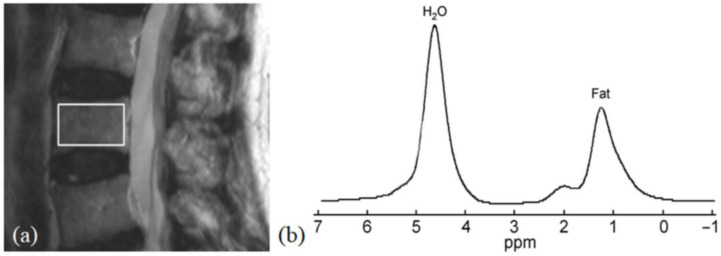
(**a**) T1-weighted sagittal MR image of the lumbar spine showing the volume of interest (VOI) defined within the L3 vertebral body. (**b**) Representative single-voxel proton MR spectroscopy (^1^H-MRS) spectrum showing normal peaks corresponding to water (H_2_O) and fat.

The width (w), depth (d), and height (h) of the L3 vertebral body were measured on MR images to define a VOI with the dimensions w/2 ∙ d/2 ∙ h/2 cm^3^ within the centre of the L3 vertebral body as previously described [8] (Figure 6). After local shimming and gradient calibration, data were acquired at a spectral bandwidth of 1000 Hz with 512 data points and 64 non-water suppressed signals using a point resolved spectroscopic sequence using as TR/TE of 3000/25 ms. The acquisition time was 2:30 min.

### 2.8. MRI Spine Image Analysis

All perfusion and spectroscopic analyses were conducted by a clinical physicist with over 20 years of experience in analysing lumbar spine perfusion and MR spectroscopy data. Analyses were performed in a blinded manner with the reader unaware of prior BMD, brain imaging measurements or other clinical data.

#### 2.8.1. Perfusion Quantification

Regions of interest (ROIs) outlining the L3 vertebral body were manually delineated on mid-axial T1-weighted images using a dedicated radiology console (ViewForum, Philips Healthcare, Best, The Netherlands) (Figure 5). Signal intensity–time curves were generated and analysed on an offline workstation (Precision 650 Workstation; Dell, Austin, TX, USA) using MATLAB R2021a (MathWorks Inc., Natick, MA, USA). Two indices derived from the time–intensity curves were derived: maximum enhancement (defined as the maximum percentage increase in signal intensity from baseline) and enhancement slope (defined as the rate of enhancement between 10% and 90% of the maximum signal intensity difference between maximum signal intensity and baseline).

#### 2.8.2. Marrow Fat Quantification

Spectral analysis was performed offline on a workstation (Precision 650 Workstation; Dell, Austin, TX, USA). Water (4.65 ppm) and lipid (1.3 ppm) peak amplitudes were measured to determine the vertebral body fat content, defined as the relative fat signal amplitude in terms of a percentage of the total signal amplitude (water and fat) (Figure 6), calculated according to the following equation: fat content = [I_fat_/(I_fat_ + I_wat_)] · 100, where I_fat_ and I_wat_ are the peak amplitudes of fat and water, respectively. No correction for relaxation losses was applied.

### 2.9. Statistical Analysis

Normality of the data was assessed using histograms and the Shapiro–Wilk test. Correlations between bone parameters (T-score, vertebral perfusion, and marrow fat content), cognitive function (HK-MoCA score), cerebral vascular measures (CCA IMT, MRA stenosis, WMH Fazekas score, WMH volume) and brain perfusion and volume (cortical and subcortical CBF, total parenchymal and hippocampal volumes) were assessed using Spearman correlation coefficients. To complement the correlation analysis and highlight differences, subjects were divided into quartiles based on L3 vertebral body perfusion and marrow fat content. Differences in cognitive, vascular, and brain imaging parameters between the lowest 25% (Q1) and the highest 25% (Q4) were assessed using independent samples *t*-tests or Mann–Whitney U tests as appropriate. All statistical tests were two-sided, with a significance threshold set at *p* < 0.05. Statistical analyses performed using SAS version 9.4 (SAS Institute Inc., Cary, NC, USA).

## 3. Results

### 3.1. Subjects

The study included a total of 99 female subjects with a mean age of 65.00 ± 5.00 years (range, 54–75 years). Mean bone mineral density DEXA T-scores were −1.58 ± 1.14 at the lumbar spine, −1.58 ± 0.93 at the femoral neck, and −1.00 ± 0.89 at the total hip region. Twenty-six (26.26%) of the 99 subjects had normal BMD, 49 (49.49%) had osteopenia, and 24 (24.24%) had osteoporosis. Baseline characteristics of the study cohort are summarised in Table 1.

### 3.2. Correlations Between Bone, Brain, Vascular and Cognitive Domains

BMD, bone perfusion and marrow content were moderately correlated with cerebral perfusion and brain volume (Table 2). Lower T-score was correlated with reduced subcortical CBF (r = 0.27, *p* = 0.031) and lower total brain parenchymal volume (r = 0.25, *p* = 0.021), where as low bone perfusion (enhancement slope) and increased marrow fat content were correlated with reduced total parenchymal volume (perfusion: r = 0.24, *p* = 0.023; marrow fat content: r = −0.26, *p* = 0.025).

Impaired cognitive function, as measured by the HK-MoCA score, correlated with both lower cortical CBF (r = 0.36, *p* = 0.005) and subcortical CBF (r = 0.31, *p* = 0.014), and with lower total parenchymal volume (r = 0.25, *p* = 0.049). Increased MRA stenosis correlated with reduced total parenchymal volume (r = −0.24, *p* = 0.027), and increased average CCA IMT correlated positively with WMH volume (r = 0.23, *p* = 0.049).

### 3.3. Inter-Correlations Within Domains

Within the bone domain, higher marrow fat content was correlated with lower T-score (r = −0.24, *p* = 0.040), and lower vertebral perfusion (maximum enhancement and enhancement slope) was inversely correlated with marrow fat content (maximum enhancement: r = −0.83, *p* < 0.001; enhancement slope: r = −0.69, *p* < 0.001), suggesting an inverse relationship between vertebral perfusion and marrow fat content.

Brain WMH measures showed internal consistency, with the WMH Fazekas score highly correlated with WMH volume (r = 0.63, *p* < 0.001). Total parenchymal volume correlated with hippocampal volume (r = 0.21, *p* = 0.047). Cortical and subcortical CBFs were strongly correlated (r = 0.80, *p* < 0.001).

### 3.4. Lowest Versus Highest Quartile Comparisons of Vertebral Perfusion and Marrow Fat

Similar trends were found when comparing subjects in the lowest and highest quartiles of L3 vertebral body perfusion and marrow fat content (Table 3). Participants in the lowest quartile of L3 vertebral body perfusion (maximum enhancement or enhancement slope) showed lower total brain parenchymal volume (76.1 ± 1.6% vs. 76.9 ± 1.1%, *p* = 0.049), lower hippocampal volume (48.7 ± 3.4% vs. 51.0 ± 3.4%, *p* = 0.029), and reduced T-scores (−1.90 ± 1.04 vs. −1.31 ± 0.84, *p* = 0.039), compared with those in the highest quartile. Participants with higher marrow fat content similarly demonstrated lower total parenchymal volume (76.1 ± 1.6% vs. 77.1 ± 1.3%, *p* = 0.034), lower hippocampal volume (47.5 ± 3.1% vs. 50.1 ± 4.4%, *p* = 0.047), and reduced T-scores (−2.08 ± 0.84 vs. −1.27 ± 0.85, *p* = 0.004), compared with those in the lowest quartile.

## 4. Discussion

To explore the potential interconnection between bone and brain health in ageing subjects, we evaluated older female subjects using a comprehensive protocol, including cognitive assessment, DEXA, carotid ultrasound, and multimodal MRI of the brain and lumbar spine.

Our findings indicate that lower BMD, decreased vertebral perfusion and increased marrow fat content are associated with reduced total brain parenchymal and/or hippocampal volumes. Correlation analyses also showed modest relationships between lower BMD and reduced subcortical brain perfusion. This suggests a link between bone health and structural brain and cerebral microvascular changes.

Osteoporosis has been previously shown to be associated with impaired bone perfusion and with increased marrow fat deposition [26,27,28], consistent with findings in our study. Increased marrow fat content reduces the metabolic demands of the bone marrow and leads to reduced marrow perfusion. Hypothalamic leptin pathways that regulate both marrow fat content and bone remodelling [29,30] may influence sympathetic nervous system activity, and potentially alter microvascular perfusion. Mechanistically, endothelial dysfunction, low-level systemic inflammation, and increased serum angiotensin-converting enzyme activity have also been observed in osteoporosis, which may further compromise perfusion [31,32].

It may be speculated that the compromised bone perfusion seen in osteoporosis may reflect a broader microvascular compromise, which may also affect cerebral circulation. Both the bone and brain are highly vascularized, and emerging evidence suggests that they share a number of common pathophysiological pathways and genetic factors. The relationship between BMD and cerebral perfusion was recently investigated in 31 elderly patients, with and without dementia, who underwent both single positron emission computerised tomography (SPECT) and DEXA examination [33]. In the analysis of 14 cerebral subregions, BMD and rCBF in the left posterior cingulate gyrus were significantly correlated in the overall population as well as in the dementia group [33]. Decreased blood flow in the left posterior cingulate gyrus was found to be an independent predictor of osteopenia [33].

Both osteoporosis and cognitive impairment are common, slowly progressive, degenerative disorders which currently are being diagnosed far too late [34]. Both diseases are silent disorders which only become manifest after irreversible clinical manifestations occur, namely fractures in osteoporosis and cognitive deficit in dementia. It is clear from the microscopic study of osteoporotic bone that features indicative of compromised bone strength are present decades before clinical bone fracture occurs [35]. Once bone mass is lost and architecture compromised, current treatments can never return it to a normal structural status. The same is likely to be true of neuronal loss and cognitive impairment. Low BMD has previously been associated with cognitive decline [6,36,37]. It would be helpful if a common link could be found between these two common debilitating disorders associated with ageing. While current therapies for bone loss and cognitive impairment are largely independent, should such a link be established, it could potentially lead to the development of shared preventive or therapeutic strategies to address both conditions simultaneously.

Although associations between BMD, bone perfusion and marrow fat with brain perfusion and volume were observed in this study, no direct relationship between bone parameters and cognitive performance was found. As expected, cognitive performance correlated with cerebral perfusion and brain volume. However, no significant associations were observed between cognitive performance and the large vessel measures of IMT or MRA, or with established WMH related to small vessel disease. 

This study has several limitations. Our study cohort only included patients clinically suspected of having mild cognitive impairment, as patients with dementia would not have been suitable candidates for comprehensive MRI and multimodal examination. Participants were older women with varying BMD. Overall, many patients were still in the early stages of either skeletal or cognitive deterioration. Consequently, the strength of observed correlations may have been reduced, and some effect sizes were small or modest. Nonetheless, even small changes in vertebral perfusion or marrow composition may potentially have cumulative effects over time, contributing to skeletal fragility or cognitive decline. Given the exploratory nature of this study, further longitudinal studies to evaluate the potential clinical relevance of these findings would be helpful. 

In this study, cognitive assessment was performed using HK-MoCA, which provides an overall measure of cognitive function, but may not evaluate subtle or domain-specific deficits. To avoid the potential nephrotoxicity of two separate contrast injections on the same day, we measured brain perfusion using ASL MRI rather than contrast-enhanced perfusion imaging. While ASL is non-invasive, it is more susceptible to noise and artefacts, and may be less sensitive than DSC MRI in some cases. Lastly, this study included exclusively female patients. It has been reported that osteoporosis and age-related bone loss are more prevalent in women, particularly postmenopausal women, due to hormonal changes that significantly affect bone metabolism. However, the potential sex-specific differences in the relationships between bone and brain physiology were not assessed.

## 5. Conclusions

In conclusion, this study shows associations between lower BMD, decreased vertebral marrow perfusion, and increased marrow fat with reduced brain parenchymal volumes and reduced brain perfusion. These findings address an important knowledge gap by providing evidence that bone-specific vascular and metabolic alterations are associated with cerebral perfusion and structural brain changes in older adults. The findings further support an interconnection between bone and cerebral health. Further studies are needed to better understand the underlying mechanisms, which could provide new insights into the shared biology of ageing.

## Figures and Tables

**Table 1:** Baseline demographic, clinical, and imaging characteristics of patients. Values are presented as mean ± standard deviation (SD) unless otherwise indicated.

Characteristic	Mean ± SD
Demographics	
Age (years)	65.00 ± 5.00
Cognitive function	
HK-MoCA score, median (IQR)	25.00 (22.00, 28.00)
DEXA examination	
Lumbar spine T-score	−1.58 ± 1.14
Femoral neck T-score	−1.58 ± 0.93
Total hip T-score	−1.00 ± 0.89
CCA intima-media thickness (IMT)	
Average CCA IMT (mm)	0.78 ± 0.17
Max CCA IMT (mm)	0.92 ± 0.24
Brain MRI—WMH	
Fazekas score	0.58 ± 0.59
Quantitative volume (mL)	0.37 ± 0.45
MRA stenosis score, frequency (%)	
0	93 (93.94%)
1	3 (3.03%)
2 or more	3 (3.03%)
ASL Perfusion	
Cortical CBF (mL/100 g/min)	33.72 ± 8.52
Subcortical CBF (mL/100 g/min)	26.63 ± 5.96
Normalised brain volume	
Total parenchymal volume (%)	76.78 ± 1.57
Hippocampal volume (%)	49.30 ± 3.62
Lumbar spine MRI—Vertebral body perfusion and fat content	
Maximum enhancement (%)	27.65 ± 7.13
Enhancement slope (%/s)	1.29 ± 0.43
Marrow fat content (%)	70.13 ± 10.71

HK-MoCA, Hong Kong version of the Montreal Cognitive Assessment; DEXA, dual-energy X-ray absorptiometry; CCA, common carotid artery; IMT, intima-media thickness; IQR, interquartile range; MRI, magnetic resonance imaging; WMH, white matter hyperintensity; MRA, magnetic resonance angiography; ASL, arterial spin labelling; CBF: cerebral blood flow.

**Table 2:** Correlation matrix of cognitive, vascular, brain and bone parameters. Values are presented as Spearman correlation coefficients (r_s_) with *p*-values in parentheses.

	T-Score	HK-MoCA Score	Average CCA IMT (mm)	WMH Fazekas Score	WMH Volume (mL)	MRA Stenosis Score	Cortical CBF (mL/100 g/min)	Subcortical CBF (mL/100 g/min)	Total Parenchymal Volume (%)	Hippocampal Volume (%)	Maximum Enhancement (%)	Enhancement Slope (%/s)	Marrow Fat Content (%)
T-score	–	–	–	–	–	–	–	–	–	–	–	–	–
HK-MoCA score	0.19 (0.110)	–	–	–	–	–	–	–	–	–	–	–	–
Average CCA IMT (mm)	0.10 (0.343)	−0.05 (0.680)	–	–	–	–	–	–	–	–	–	–	–
WMH Fazekas score	0.14 (0.187)	−0.12 (0.289)	0.08 (0.485)	–	–	–	–	–	–	–	–	–	–
WMH volume (mL)	0.002 (0.982)	−0.25 (0.053)	0.23 (0.049) *	0.63 (<0.001) ***, †††	–	–	–	–	–	–	–	–	–
MRA stenosis score	−0.09 (0.396)	−0.21 (0.074)	0.03 (0.747)	0.06 (0.579)	0.11 (0.288)	–	–	–	–	–	–	–	–
Cortical CBF (mL/100 g/min)	0.19 (0.132)	0.36 (0.005) **	0.03 (0.832)	−0.06 (0.662)	−0.01 (0.962)	0.14 (0.271)	–	–	–	–	–	–	–
Subcortical CBF (mL/100 g/min)	0.27 (0.031) *	0.31 (0.014) *	0.03 (0.809)	−0.03 (0.829)	0.12 (0.363)	0.03 (0.808)	0.80 (<0.001) ***, †††	–	–	–	–	–	–
Total parenchymal volume (%)	0.25 (0.021) *	0.25 (0.049) *	0.01 (0.922)	−0.05 (0.659)	−0.09 (0.405)	−0.24 (0.027) *	0.17 (0.176)	0.18 (0.168)	–	–	–	–	–
Hippocampal volume (%)	0.12 (0.262)	−0.03 (0.797)	−0.03 (0.821)	0.18 (0.101)	0.13 (0.244)	−0.17 (0.104)	0.11 (0.387)	0.12 (0.347)	0.21 (0.047) *	–	–	–	–
Maximum enhancement (%)	0.12 (0.261)	−0.08 (0.520)	0.08 (0.472)	0.05 (0.629)	−0.08 (0.461)	−0.03 (0.754)	0.12 (0.350)	0.18 (0.157)	0.17 (0.111)	0.20 (0.063)	–	–	–
Enhancement slope (%/s)	0.13 (0.208)	−0.06 (0.604)	0.15 (0.178)	0.09 (0.374)	0.00 (0.972)	0.07 (0.491)	0.14 (0.277)	−0.13 (0.357)	0.24 (0.023) *	−0.20 (0.091)	0.71 (<0.001) ***, †††	–	–
Marrow fat content (%)	−0.24 (0.040) *	−0.02 (0.892)	−0.10 (0.426)	−0.12 (0.300)	0.06 (0.639)	0.05 (0.685)	−0.08 (0.582)	−0.13 (0.357)	−0.26 (0.025) *	−0.20 (0.091)	−0.83 (<0.001) ***, †††	−0.69 (<0.001) ***, †††	–

* *p* < 0.05; ** *p* < 0.01; *** *p* < 0.001; ††† *p*_adj_ < 0.001 after Bonferroni correction; HK-MoCA, Hong Kong Montreal Cognitive Assessment; CCA, common carotid artery; IMT, intima-media thickness; WMH, white matter hyperintensity; MRA, magnetic resonance angiography; CBF, cerebral blood flow.

**Table 3:** Comparison of brain, cognitive, and bone parameters between extreme quartiles of L3 vertebral body perfusion (maximum enhancement, enhancement slope) and marrow fat content. Subjects in the lowest (Q1) and highest (Q4) quartiles of L3 vertebral body perfusion (maximum enhancement, enhancement slope) and marrow fat content were compared. Only parameters showing statistically significant differences (*p* < 0.05) are shown.

	Lowest Quartile (Mean ± SD)	Highest Quartile (Mean ± SD)	*p*-Value
**Maximum enhancement (%)**			
Total parenchymal volume (%)	76.05 ± 1.64	76.91 ± 1.11	0.049
Hippocampal volume (%)	48.65 ± 3.40	50.96 ± 3.35	0.029
**Enhancement slope (%/s)**			
T-score	−1.90 ± 1.04	−1.31 ± 0.84	0.039
**Marrow fat content (%)**			
T-score	−1.27 ± 0.85	−2.08 ± 0.84	0.004
Total parenchymal volume (%)	77.14 ± 1.28	76.07 ± 1.62	0.034
Hippocampal volume (%)	50.07 ± 4.38	47.45 ± 3.05	0.047

*p* < 0.05 was considered statistically significant; SD, standard deviation.

## Data Availability

The data supporting this study are not publicly available due to institutional regulations and patient privacy considerations.

## Supplementary Materials

The following supporting information can be downloaded at https://www.mdpi.com/article/10.3390/diagnostics16040529/s1, Table S1: Acquisition details for MRI brain sequences.

## Abbreviations

The following abbreviations are used in this manuscript:

AAL	Automated Anatomical Labelling
ANOVA	Analysis of Variance
ASL	Arterial Spin Labelling
BMD	Bone Mineral Density
BMDa	Areal Bone Mineral Density
CBF	Cerebral Blood Flow
CCA	Common Carotid Artery
DCE	Dynamic Contrast-Enhanced
DEXA	Dual-Energy X-ray Absorptiometry
DSC	Dynamic Susceptibility Contrast
EPI	Echo-Planar Imaging
FOV	Field of View
HK-MoCA	Hong Kong Version of Montreal Cognitive Assessment
IMT	Intima-Media Thickness
ISMRM	International Society for Magnetic Resonance in Medicine
MCI	Mild Cognitive Impairment
MRA	Magnetic Resonance Angiography
MRS	Magnetic Resonance Spectroscopy
NSA	Number of Signal Averages
pCASL	Pseudocontinuous Arterial Spin Labelling
PLD	Post-Labelling Delay
ROI	Region of Interest
SENSE	Sensitivity Encoding
SPM	Statistical Parametric Mapping
SPECT	Single Photon Emission Computed Tomography
TE	Echo Time
TFE	Turbo Field Echo
TOF	Time-of-Flight
TR	Repetition Time
TSE	Turbo Spin Echo
VOI	Volume of Interest
WMH	White Matter Hyperintensity
