# Supplementary material for: Poor Bone Health Associated with Reduced Cerebral Perfusion and Brain Volume in Older Adults
Source: Diagnostics (Basel). 2026 Feb 10;16(4):529. doi: 10.3390/diagnostics16040529 (PMC12939405; doi:10.3390/diagnostics16040529)
Supplement: Supplementary file 1 [file diagnostics-16-00529-s001.zip › Supplementary Table S1.pdf]

Supplementary Table S1. Acquisition details for MRI brain sequences

Parameter	3D T1-weighted	Axial T2-weighted	3D FLAIR	3D TOF-MRA	pCASL
Sequence type	TFE	Fat-suppressed TSE	3D TSE (VISTA)	TOF	Single-shot EPI readout
Plane	Sagittal	Axial	Sagittal	Axial	Axial
TR (ms)	7.4	5472	8000	25	4000
TE (ms)	3.4	80	332	3.5	14
Flip angle (°)	8	–	–	20	90
FOV (mm^3)	250 × 250	230 × 207	230 × 230	180 × 180 × 84	240 × 240 × 119
Slice thickness (mm)	0.6	5	0.55	0.7	7
Matrix	240 × 240	512 × 350	208 × 208	600 × 600	80 × 79
Inversion time (ms)	–	–	2400	–	–
Inversion delay (ms)	–	–	220	–	–
Acquisition time	5:07 min	2:56 min	5:52 min	4:50 min	5:27 min
Additional parameters	–	Echo train length = 18; SENSE factor = 1.5	–	–	Labelling: Hanning-shaped RF pulses (flip angle 18°, duration 0.5 ms, inter-pulse 0.5 ms, label duration 1650 ms); Background suppression: pre-saturation and inversion pulses at 1680 & 2830 ms; PLD: 1525ms; Label-control pairs = 38; SENSE factor = 2.5

DCE, Dynamic Contrast-Enhanced; FLAIR, Fluid-Attenuated Inversion-Recovery; FOV, Field of View; MRA, MR Angiography; NSA, Number of Signal Averages; pCASL, post-labelling delay (PLD); Pseudocontinuous Arterial Spin Labelling; SENSE, Sensitivity Encoding; TFE, Turbo Field Echo; TSE, Turbo Spin Echo; TOF-MRA, Time-of-Flight MR Angiography; TR, Repetition Time; TE, Echo Time.
